# Unveiling the Psychological Consequences of Illness Perception in Pediatric Multiple Sclerosis: A Parent–Child Study

**DOI:** 10.3390/children11080929

**Published:** 2024-07-31

**Authors:** Roy Aloni, Gaya Asher, Amichai Ben-Ari, Shay Menascu

**Affiliations:** 1 Department of Psychology, Ariel University, Ariel 4077625, Israel; roya@ariel.ac.il (R.A.); gayaa@ariel.ac.il (G.A.); baamichai@gmail.com (A.B.-A.); 2 Multiple Sclerosis Center, Sheba Medical Center, Tel Hashomer, Ramat Gan 5262160, Israel; 3 Tel-Aviv School of Medicine, Tel-Aviv University, Tel Aviv 6139001, Israel

**Keywords:** multiple sclerosis, pediatric, children, illness perception, anxiety, depression, dyadic, comorbidity

## Abstract

Background: Previous research has emphasized the significant role of illness perception in chronic diseases, including Multiple Sclerosis. Limited research has been conducted on exploring illness perception in Pediatric Onset Multiple Sclerosis (POMS), parental illness perception, and the impact of differences in their illness perceptions on the emotional well-being of the child. Method: This study included 65 dyads of children aged 10–17 and their parents, divided into the following two groups: (I) 32 dyads of children with POMS and their parents; and (II) 33 dyads of healthy children and their parents. Results: Overall, 73.1% and 43.8% of the children with POMS met the criteria for probable anxiety and depression, respectively, compared to 27.3% and 0% of the healthy children. Differences were found between the dimensions of illness perception in the POMS children and their parents, in the areas of consequences, personal control, identity, and control factors. Multinomial Logistic Regression indicated that differences in child–parent illness perception increased the likelihood of comorbid anxiety and depression by 37%. Discussion: These findings underscore the importance of alignment between children with POMS and their parents in illness perception. Healthcare providers should prioritize interventions that address illness perceptions and be mindful of the potential impact on depression and anxiety comorbidity.

## 1. Introduction

Multiple sclerosis (MS) is a chronic neurological disease characterized by diffuse demyelination of the white matter in the central nervous system (CNS) [1]. The immune system attacks the myelin that wraps the nerve cell extensions and leads to neurodegeneration and axonal injury processes in the CNS. The damage caused is of different degrees of severity and in different brain areas thus affecting the onset of the disease in different ways [2,3]. MS is the leading cause of neurological disability in young people [4]. Typically, its onset is observed during the second to fourth decades of life. In instances involving individuals under the age of 18, the condition is referred to as Pediatric-Onset Multiple Sclerosis (POMS) [5], which constitutes approximately 3–5% of individuals with MS [6]. While MS in adults shows a distinct female dominance, the sex ratio of POMS occurs almost equally between the sexes before puberty [7]. Moreover, POMS is linked with higher relapse rates, rapid MRI lesion accrual early in the disease course, and worse cognitive outcomes and physical disability in the long term compared to the adult-onset disease [8]. Common symptoms of POMS may be similar to those present in adult MS, with visual, sensory, motor, and coordination impairments, bladder and bowel problems [9], as well as cognitive difficulties in attention, processing speed, visual–motor skills, and language [10,11,12,13].

Alongside these challenges, children with POMS face a significant risk of psychiatric comorbidities [14], such as depression, which occurs in up to 50%, and anxiety disorders in 64% [11,13]. Based on parental reports, a discernibly higher incidence of depressive symptoms was noted among the children with demyelinating disorders when compared to the healthy controls (28.6% vs. 10.9%; *p* = 0.008) [15]. Subsequent inquiries corroborated the heightened prevalence of depression in the children with MS relative to the healthy control group (22.7% vs. 11.4%; *p* = 0.014) [16]. Emotional disturbances in POMS carry significant ramifications, with attention to depression and anxiety having been shown to exert adverse effects on quality-of-life scores, as well as on factors such as fatigue, disability, and disease duration. Additionally, a systematic review indicates that comorbid anxiety and depression are commonly seen in children, and might be even higher among children and adolescents when compared to adults [17]. This pattern is also significant in the case of children with POMS [18], and even more common compared to non-brain-related chronic diseases [19]. The potential prediction of comorbidity leading to a decline in overall functioning, including negative impacts on academic performance and increased familial conflict [17], underscores the importance of thoroughly examining it within the context of chronic illnesses such as POMS.

Despite the significant impact of emotional difficulties in this population, research in this area is relatively limited and lacks a comprehensive understanding of the various factors influencing the emotional well-being of children with POMS. One of the crucial factors is the perception of illness, a subject that has been widely studied in the last decade among patients with chronic disease and MS and refers to people’s cognitive and emotional representations of an illness [20,21]. Leventhal’s self-regulation model (SRM) posits that people experiencing illness formulate personalized representations of their illness. These perceptions are central in shaping coping strategies and emotional reactions. The dimensions of these representations include the representations or beliefs about illness (identity), assumptions about the duration or course (timeline), causes and agents (causes), beliefs about the impact of the disease on daily life (consequences), and the perceived ability to exert control over the situation through personal actions and medical therapies, (control) [22,23]. Additional dimensions, as suggested by Broadbent et al. and Moss-Morris et al. [24,25], include understanding the disease (coherence) and its emotional effects (emotional representations). Illness perceptions play a pivotal role in shaping individuals’ responses to a disease’s challenges and influencing their psychological adjustment to the condition [26]. It has been observed that negative illness perceptions often report reduced satisfaction following medical consultations, exhibit poor adherence to prescribed treatment regimens, experience compromised coping mechanisms, and manifest poorer psychophysical well-being [27].

To the best of our knowledge, limited research has investigated facets of illness perception in POMS. Current studies indicate that a child’s skepticism about treatment efficacy, coupled with a diminished sense of personal control and inadequate illness management, increases the likelihood of experiencing heightened social isolation. Such isolation, in turn, correlates with the manifestation of symptoms related to depression and anxiety [28,29]. Traditionally, discussions on illness perceptions have primarily centered on the patient’s perspectives [30]. However, in the case of pediatric patients, it is imperative to also explore the parental perceptions of illness. This is particularly pertinent as parents often play a crucial role in assimilating information from healthcare professionals and making decisions about the care of their child. Furthermore, negative perceptions held by parents regarding their child’s illness have the potential to compound their distress [24,31].

Previous research on chronic diseases in children examined the patients’ and caregivers’ illness beliefs, considering the collective impact on adjustment and quality of life. Special emphasis was placed on evaluating the level of congruence, distinguishing between similarity and dissimilarity, in the representations held by patients and their caregivers. When discrepancies were present, the caregivers expressed more pessimistic perspectives on elements such as illness identity, chronicity, consequences, and emotional representations [32,33,34]. Moreover, positive congruent perceptions were associated with better adjustment, while congruent negative perceptions correlated with poorer adjustment [34]. In line with these studies, Bassi et al. [35] found that compared to adult patients with MS, caregivers held more pessimistic views about MS identity and emotional representations and a more optimistic perception of personal control, which could arise from an effort to reduce the uncertainty associated with an unpredictable condition like MS.

The current investigation sheds light on the nuances of illness perception among children with POMS. Existing knowledge about the self-perception of children with MS is limited. Furthermore, the distinct perspectives on illness perceptions in cases where parents act as the primary caregivers for children with POMS remain unexplored. Additionally, we aim to examine the correlation between the level of agreement between children and their parents regarding this illness and the psychological distress experienced by children, with a particular focus on comorbid depression and anxiety.

## 2. Materials and Methods

### 2.1. Participants

The current study included 65 dyads of children and their parents, divided into two groups as follows: (I) 32 parent–child dyads with children diagnosed with POMS and their parents (POMS group); and (II) 33 parent–child dyads with healthy children without a diagnosis of MS and their parents (HC group). The two groups of children matched in sociodemographic parameters; however, there was a significant difference in the sex and education of the parents. Healthy control parents were mostly female and had higher academic education levels than POMS parents. See Table 1 for more details.

The criteria for inclusion were diagnosis based on the revised McDonald’s criteria [36], age under 18 years, and an Expanded Disability Status Scale (EDSS) score under 5 [37]. Criteria for exclusion included other neurological or medical diagnoses, alcohol or substance use, ongoing relapse, or steroid treatment in the 90 days preceding assessment.

### 2.2. Measures

#### 2.2.1. Sociodemographic and Medical Information

The sociodemographic and medical information collected included gender, age, educational levels, socioeconomic status as well as disease duration, disease-modifying treatments (DMTs), and EDSS for POMS group. The EDSS is a clinician-administered assessment scale, the most accepted index for measuring the severity of MS among all ages [38], based on an examination by a neurologist. The scale ranges from 0 (normal neurological status) to 10 (death due to MS) in 0.5 unit increments that represent higher levels of disability. EDSS steps 1.0 to 4.5 refer to patients who can walk without assistance and are based on measures of impairment in eight functional systems, namely pyramidal, cerebellar, brainstem, sensory, bowel and bladder, visual function, cerebral functions, and cognition. EDSS steps 5.0 to 9.5 are defined mainly by walking impairment [37].

#### 2.2.2. Depression and Anxiety Symptoms

Depression and anxiety symptoms were measured among the children in both groups via the Hospital Anxiety and Depression Scale (HADS) [39]. The HADS is a self-assessed questionnaire consisting of 14 multiple-choice items (with a Likert scale range of 0–3) probing symptoms of anxiety (7 items) and depression (7 items). Generally, scores of 11 and above indicate clinical distress. For MS, a score of 8 or above was recommended since it was found to be an accurate indicator of major depression (90% sensitivity, 87% specificity) and generalized anxiety disorder (88.5% sensitivity, 81% specificity) [40]. HADS is widely used among POMS patients and has high internal reliability among this population on the anxiety scale (Cronbach’s α = 0.80) and on the depression scale (Cronbach’s α = 0.81) [41]. In the current study, good internal reliability was found for depression and anxiety scales (Cronbach’s α = 0.79 and 0.89, respectively).

#### 2.2.3. Illness Perception

Illness perceptions were assessed using the Brief Illness Perception Questionnaire (B-IPQ) [25] for children with POMS and their parents who were asked about their children’s illness. The questionnaire evaluates eight dimensions with a single item for each dimension, covering consequences of the disease, timeline, personal control, treatment control, identity, concern, illness coherence, and emotional response. Responses are scored on a Likert scale from 0 to 10. To maintain consistency, positive scores in items dealing with self-control, treatment control, and understanding of the disease were reversed to negative scores. The total score was computed by summing up all items with a reverse scoring of items 3, 4, and 7 in a range of 0–80, with higher scores indicating more severe and negative disease perceptions. The B-IPQ has been validated and proven to be reliable in various diseases, with good psychometric properties demonstrated in a meta-analysis [42]. Additionally, the questionnaire has shown good validity and reliability among people with MS (Cronbach’s α = 0.71–0.76) [43]. In the current study, the internal consistency was good for children with POMS and their parents (Cronbach’s α = 0.68 and 0.72, respectively).

### 2.3. Procedure

In both groups, data were prospectively collected during a face-to-face meeting. For the POMS group, data were gathered during a regular visit to the MS department of the tertiary medical center. Participants completed self-report questionnaires as part of a comprehensive evaluation conducted by the department. The children were asked to independently fill out the questionnaires in the presence of a research assistant who could provide clarification if needed. The study received ethical approval from the Ethics Committee [5596-08]. As part of the initial evaluation, patients underwent tests to rule out other underlying diseases such as vasculitis, rheumatological diseases, and coagulation disorders, as well as vision and blood tests and a specialized MRI for MS diagnosis. Additionally, all patients received a clinical neurological examination (EDSS) by a qualified and experienced pediatric neurologist.

The HC group was recruited using social media, university mailing lists, and neighborhood WhatsApp groups. Once the parents agreed to their child’s participation, a face-to-face meeting was scheduled. Before the parent and child completed the questionnaire, the parent read and signed the informed consent form. This part of the study was approved by the Institutional Ethics Board [AU-SOC-RA-20210811].

### 2.4. Statistical Analysis

In the preliminary analysis phase, the data’s aptitude for normal distribution was verified through the Kolmogorov–Smirnov test. Comparisons were made regarding the participants’ characteristics by implementing the appropriate statistical tests—Student’s *t*-test, Mann–Whitney U test, and Chi-squared test—based on the data distribution. These tests were used to draw comparisons between children diagnosed with MS and their healthy counterparts (HC), with the characteristics under scrutiny encompassing a spectrum of sociodemographic variables including the age and gender of parents and children, parental education levels, and family income. Similarly, clinical manifestations of anxiety and depression were contrasted across the two groups. In addition, a series of Wilcoxon signed-rank tests was employed to investigate discrepancies in illness perception between the parents and children within the group of children diagnosed with MS. In the concluding phase of the analysis, a multinomial logistic regression model was implemented within the MS-diagnosed children cohort. The primary aim was to ascertain the likelihood of developing anxiety or depressive disorders, in addition to assessing the potential comorbidity of these conditions. This regression model considered differences in illness perceptions between the parent and children, whilst adjusting the Expanded Disability Status Scale (EDSS) and disease duration. Continuous variables are presented as mean ± standard deviation (SD), and categorical variables are expressed in counts (percentages). All statistical tests employed were two-tailed, and statistical significance was determined at a threshold of *p* ≤ 0.05. Data analysis was performed using the IBM SPSS Statistics software, version 27.

## 3. Results

### 3.1. Differences in Emotional Distress between Children with POMS and HC

Two chi-squared tests of independence were conducted to investigate the differences in emotional distress (anxiety and depression levels) between children with POMS and those with HC. For anxiety, the chi-squared test of independence indicated a significant association between the groups and anxiety, χ^2^(1, n = 65) = 16.84, *p* < 0.001, rc = 0.51. The children with POMS (78.1%) were more likely to experience clinical levels of anxiety compared to those with HC (27.3%). Similarly, there was a significant association between the groups and depression, χ^2^(1, n = 65) = 18.40, *p* < 0.001, rc = 0.53. The children with POMS (43.8%) were more likely to exhibit clinical levels of depression when compared to the HC group (0%). Furthermore, continuous variables for anxiety and depression were calculated. The mean and standard deviation for anxiety were 10.34 (SD = 4.35) in the group of children with MS and 4.64 (SD = 3.50) in the HC group. For depression, the mean and standard deviation were 6.66 (SD = 3.33) in the MS group, and 1.88 (SD = 1.93) in the HC group.

### 3.2. Illness Perception among Children with POMS and Their Parents

**Figure 1:**
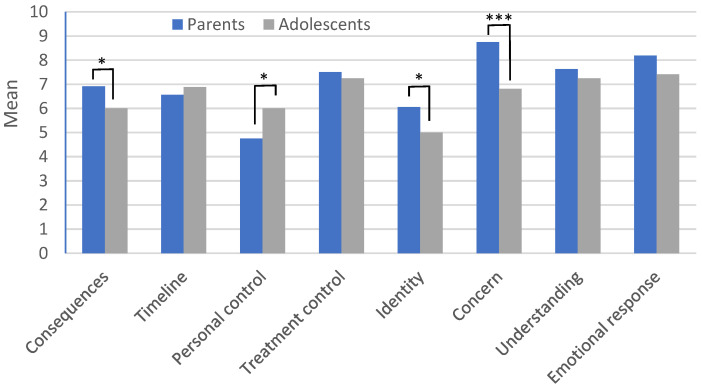
Differences in illness perceptions’ dimensions between children with POMS and their parents. * *p* < 0.05; *** *p* < 0.001.

Given the unique characteristics and challenges of the children with MS group, a series of nine Wilcoxon signed-rank tests were conducted specifically within this group to compare parent and child illness perceptions. A summary of the Wilcoxon signed-rank test results, including the Z values, mean, and standard deviation of illness perception scores for parents and children, are presented in Table 2 and Figure 1. The analysis showed significant differences in the total scores between the parents and children in the following four dimensions: consequences, personal control, identity, and concern. So, generally, parents have a more negative illness perception; they perceive the illness as having a more severe identity and consequences and are more concerned about it than their children. Conversely, children reported higher personal control over their illness than their parents.

### 3.3. The Effect of Disease Characteristics and Illness Perception on Psychopathology among Children with POMS

Moreover, a multinomial logistic regression analysis was conducted to assess the effect of EDSS and disease duration on the differences in illness perception between parents and children on the nominal dependent variable with the following three levels: (1) no disorder, (2) either anxiety or depression, and (3) comorbidity of both disorders. The overall model was found to be statistically significant (χ^2^(6) = 22.74, *p* < 0.001), indicating that the model could distinguish between respondents who reported no disorder, either anxiety or depression, or both disorders. The model explained between 50.9% and 58% (Cox and Snell R^2^ = 0.509; Nagelkerke R^2^ = 0.58) of the variance in disorder status, and correctly classified 56.3% of cases. Both Pearson’s and Deviance goodness-of-fit measures (χ^2^(56) = 48.10, *p* = 0.764 and χ^2^(56) = 44.19, *p* = 0.873, respectively) validated a satisfactory model fit to the observed data. As indicated in Table 3, the illness perception difference between child and parent was found to be a statistically significant predictor (χ^2^(2) = 14.96, *p* = 0.001) of the dependent variable. However, EDSS (χ^2^(2) = 1.38, *p* = 0.503) and disease duration (χ^2^(2) = 3.41, *p* = 0.182) were not statistically significant predictors of the outcome variable.

These results indicate that for the comparison between children with Both Disorders and No Disorder, the Illness Perception Difference was a significant predictor. Specifically, while holding EDSS and Disease Duration constant, for each one-unit increase in Illness Perception Difference, the odds of being in the Both Disorders category increased by 37% (OR = 1.37). Similarly, when comparing the children with Both Disorders and Anxiety/Depression, the Illness Perception Difference was also found to be significant. For each one-unit increase in the Illness Perception Difference, the odds of being in the Both Disorders category increased by 28% (OR = 1.28). These results suggest that while Illness Perception Difference does not distinguish between No Disorder and Anxiety/Depression, it does significantly increase the odds of having both anxiety and depression compared to either No Disorder or only Anxiety/Depression when EDSS and Disease Duration are accounted for.

## 4. Discussion

This study represents the first examination of illness perception and its differences between children with POMS and their parents. Additionally, it uniquely connects these findings to existing knowledge about anxiety and depression within this population, with attention to the comorbidity of these conditions. The results offer valuable insights into the psychological and emotional challenges faced by young patients with POMS and their families, highlighting the critical role of illness perception in shaping emotional distress and overall mental health.

The study revealed significant differences in emotional distress, specifically anxiety and depression levels, between children with POMS and healthy controls. Children with POMS exhibited higher rates of clinical anxiety (78.1%) compared to healthy controls (27.3%) and higher rates of clinical depression (43.8%) compared to healthy controls (0%). These results are consistent with prior research indicating heightened psychiatric comorbidities in children with POMS. Studies have shown that depression occurs in up to 50% and anxiety disorders in 64% of children with POMS [11,13]. The elevated levels of emotional distress in the children with POMS compared to the healthy controls can be attributed to the significant psychological and physical burden of managing a chronic and unpredictable disease from a young age. Children with MS often experience chronic stress due to the lifelong management of their condition. In addition to dealing with disease-related symptoms and physical disabilities, they also face stressful situations such as hospitalization and emotional distress [44]. Furthermore, they encounter developmental challenges, including identity formation and peer relationships [45]. The findings of the current essay, supported by previous findings, suggest that children with POMS are at a significantly greater risk of experiencing emotional distress than their healthy peers.

Another goal of this study is to focus on illness perception among POMS. Illness perceptions affect the adjustment to neurological diseases [46], including MS [35,47]. As indicated by Jopson and Moss-Morris [48], illness identity, perceptions of a chronic timeline and severe consequences, attributions to psychological causes, low illness coherence, and diminished personal control are factors associated with elevated levels of anxiety and depression. Recent research has found that illness concerns and emotional representation emerged as the most influential predictors of psychological distress in individuals diagnosed with MS [21]. The current study explored differences in illness perception between the children with POMS and their parents and found significant differences in total score and several dimensions such as consequences, personal control, identity, and concern. Overall, the parents generally perceived the illness of their children more negatively; they perceived more severe identity and consequences and were more concerned about it than their children. Conversely, the children reported higher personal control over their illness than their parents. Similar discrepancies have been observed in studies involving other chronic conditions such as diabetes [33,34], rheumatic disease [31], and cancer [49], where parents’ perceptions often highlight greater concern and perceived impact than their children’s.

In addition, the present study aimed to test the hypothesis that perception gaps of the illness between children and their parents, along with disease variables, can affect the child’s psychological adjustment. According to our hypotheses, the difference in illness perception significantly predicted the odds of being in the group with comorbidity of depression and anxiety. Each one-unit increase in illness perception difference increased the odds by 37% (OR = 1.37). However, illness perception differences did not distinguish between children with no disorder and children with only one condition (Anxiety or Depression). This finding underscores the importance of illness perception in managing chronic illnesses and its impact on psychological outcomes [27,28]. Previous research has suggested that congruent positive perceptions between parent and child correlated with better adjustment, while congruent negative perceptions correlated with poorer adjustment [36]. An increased disparity in illness perception between parent and child was linked to heightened child distress, potentially manifesting in additional and profound symptoms, which were found to relate to comorbid depression and anxiety, such as negative self-evaluation and discouragement towards the illness [16]. However, our study emphasizes the predictive power of illness perception differences, with a focus on the comorbidity of anxiety and depression, which is well recognized as having negative effects on the quality of life in MS [50].

Our findings provide a more nuanced understanding by highlighting the subjective experiences as reflected in illness perception and its effect on emotional distress, above and beyond disease characteristics. In our model, disease severity (as measured by EDSS) and disease duration were insignificant predictors. The literature on this topic is mixed. Previous studies in adults with MS showed a significant relation between EDSS and depression and anxiety [51,52], and others found only partial relations between anxiety and depression and neurological disability [53,54], while others found no association at all [55,56]. Among children with POMS, the picture is similar, and studies have highlighted mixed results regarding the predictive power of disease severity and duration on psychological outcomes [11,57]. Our findings support the possible explanation that psychological distress in POMS may be more strongly influenced by cognitive and emotional factors, such as illness perception, rather than physical disability or disease duration alone. Moreover, due to the lack of evidence among POMS, this finding is essential for future studies to investigate the interplay between psychological distress and disease characteristics.

The findings of this study have several clinical implications. First and foremost, healthcare providers should prioritize interventions that address illness perceptions among children with POMS and their parents. According to the evidence accumulated from the literature, various influences and attributions have been found among family members of children with POMS [58]. So, systematic family therapy, which was found effective, should be used in the case of chronic illnesses [59], with integration of cognitive-behavioral approaches, as suggested by Petrie et al. [27]. This could be effective in modifying maladaptive illness perceptions and improving psychological outcomes. Clinicians should focus on the proper diagnosis with close attention to comorbid conditions and then plan the proper treatment to ensure that the children with POMS receive appropriate support, which can lead to improved academic and social outcomes.

### Limitations and Future Research

One of the primary limitations of the current study is the relatively small sample size of 65 dyads. Although children with POMS are considered a relatively small and unique population, this limitation can impact the generalizability of the findings. Moreover, while children in both groups matched with age and sex, there were differences in sex and education between the parents, a fact that should be considered carefully when interpreting parent reports. In addition, our small sample size limited our ability to explore possible differences in perception between the mother and father about their child’s illness and for a more precise conclusion on their impact on the child’s adjustment. Future studies should aim to recruit a larger and more diverse sample with international collaboration to improve the generalizability of the findings, with attention to the potentially different impacts of fathers and mothers on their children. Secondly, the cross-sectional design of this study limits the ability to draw causal inferences. While significant associations between illness perception and emotional distress were identified, it is not possible to determine the directionality of these relationships. Longitudinal studies would offer more robust insights into the causal pathways and the temporal dynamics of illness perception and psychological outcomes. Future research should incorporate longitudinal designs to better understand the evolution of illness perception and its impact over time. Furthermore, incorporating objective measures and controlling for a broader range of confounding variables, such as socio-economic status and family dynamics, could provide a more comprehensive understanding of the factors influencing psychological outcomes in POMS.

## 5. Conclusions

In conclusion, the findings of this study highlight the critical role of psychological support and tailored interventions for children with POMS and their families. Addressing the emotional and cognitive aspects of the illness and fostering better communication and understanding between children and their parents, alongside support groups and the attending of healthcare staff, are essential steps in improving the quality of life and psychological outcomes for these young patients. Future research should continue to explore these dynamics, addressing the limitations and expanding on the findings to develop comprehensive care strategies for this vulnerable population.

## Figures and Tables

**Table 1:** Sociodemographic and medical characteristics.

Characteristics	POMS (n = 32)	HC (n = 33)	*p*
Age, mean (SD), range	15.30 (2.00), 10–17	14.82 (2.13), 10–17	0.328
Female, n (%)	15 (46.9%)	17 (51.5%)	0.708
Parent age, mean (SD)	45.34 (5.11)	45.94 (6.16)	0.673
Female parent, n (%)	18 (56.3%)	32 (97%)	<0.01 **
Parent education			
Elementary to high school, n (%)	18 (64.3%)	10 (35.7%)	0.017 *
Academic, n (%)	12 (34.3%)	23 (65.7%)	
Family income			
Below average, n (%)	6 (18.8%)	4 (12.5%)	0.373
Average, n (%)	19 (59.4%)	16 (50%)	
Above average, n (%)	7 (21.9%)	12 (37.5%)	
Age at onset, mean (SD)	11.16 (3.76)	-	
MS-duration in months, mean (SD)	37.31 (25.83)	-	
EDSS score, mean (SD)	1.14 (0.63)	-	
Disease modifying drug, n (%)	28 (87.5%)	-	

Note. POMS = Pediatric Onset Multiple Sclerosis, HC = Healthy children; EDSS = Expanded Disability Status Scale. * *p* < 0.05; ** *p* < 0.01.

**Table 2:** Wilcoxon signed-rank tests comparing parent and children illness perception.

		Parents		Children			
Dimension	Items	M	SD	M	SD	Z	*p*
Consequences	How much does your/child illness affect your/child life?	6.91	2.70	6.00	3.36	−1.87	0.031 *
Timeline	How long do you think your/child’s illness will continue?	6.56	2.60	6.88	3.01	−0.64	0.268
Personal control	How much control do you feel you have over your/child illness?	4.75	2.62	6.00	2.48	−2.34	0.019 *
Treatment control	How much do you think your treatment can help your/child) illness?	7.50	1.44	7.25	2.53	−0.04	0.967
Identity	How much do you/your child experience symptoms from your/his illness?	6.06	2.82	5.00	2.95	−2.00	0.022 *
Concern	How concerned are you about your/child illness?	8.75	1.69	6.81	3.17	−3.50	<0.001 ***
Understanding	How well do you feel you understand your/child illness?	7.63	1.56	7.25	2.41	−0.53	0.595
Emotional response	How much does your/child illness affect you emotionally?	8.19	2.31	7.41	2.85	−1.31	0.099
Total score		46.6	8.62	41.8	14.57	−2.12	0.034 *

Note. The statements differ for child/parent; * *p* < 0.05; *** *p* < 0.001.

**Table 3:** Multinomial logistic regression analysis predicting disorder status based on illness perception difference, EDSS, and disease duration.

	B	SE	Wald χ^2^	OR	*p*
No Disorder vs. Anxiety/Depression					
EDSS	−0.12	0.90	0.02	0.89	0.892
Disease Duration	−0.03	0.02	2.01	0.97	0.157
Illness Perception Difference	0.07	0.05	1.73	1.07	0.189
No Disorder vs. Both Disorders					
EDSS	−1.18	1.24	0.89	0.31	0.345
Disease Duration	−0.05	0.03	2.53	0.95	0.112
Illness Perception Difference	0.32	0.13	5.73	1.37	0.017 *
Anxiety/Depression vs. Both Disorders					
EDSS	−1.05	0.98	1.16	0.35	0.281
Disease Duration	−0.02	0.03	0.52	0.98	0.469
Illness Perception Difference	0.25	0.12	4.08	1.28	0.043 *

* *p* < 0.05.

## Data Availability

The data presented in this study are available on request from the corresponding author due to privacy and ethical restrictions.
